# Rodent adapted marburg viruses are lethal in ferrets

**DOI:** 10.1038/s44298-025-00147-4

**Published:** 2025-09-08

**Authors:** Zachary Schiffman, Lauren Garnett, Kaylie N. Tran, Jonathan Audet, Kevin Tierney, Kim Azaransky, Shihua He, Logan Banadyga, James E. Strong

**Affiliations:** 1 Special Pathogens Program, National Microbiology Laboratory Branch, Public Health Agency of Canada, Winnipeg, MB Canada; 2 Department of Medical Microbiology and Infectious Diseases, University of Manitoba, Winnipeg, MB Canada; 3 Department of Pediatrics and Child Health, University of Manitoba, Winnipeg, MB Canada

**Keywords:** Microbiology, Virology, Marburg virus

## Abstract

Ferrets are highly susceptible to infection with several orthoebolaviruses, including Ebola virus (EBOV), yet they are refractory to infection with the orthomarburgviruses, Marburg virus (MARV) and Ravn virus. This study sought to investigate the pathogenicity of rodent-adapted MARV in ferrets. Challenge with guinea pig-adapted (GPA)-MARV resulted in uniform lethality among ferrets, whereas challenge with mouse-adapted (MA)-MARV resulted in partial lethality. Ferrets challenged with GPA-MARV manifested clinical signs of filovirus disease, including a petechial rash. These animals supported high levels of viral replication and exhibited coagulation abnormalities exemplified by thrombocytopenia, a feature absent among ferrets challenged with GPA-EBOV. A dysregulated immune response and hematological perturbations were also observed among GPA-MARV-challenged ferrets. Lastly, several genome mutations appeared in GPA-MARV following ferret challenge. This study provides insight into the pathogenesis of MARV and represents the first report and characterization of a lethal Marburg virus ferret model.

## Introduction

The domestic ferret (*Mustela putorious furo*) has gained attention in recent years as an animal model for characterizing filovirus pathogenesis and evaluating the efficacy of candidate vaccines and countermeasures^1^. Indeed, ferrets share many similar anatomical, metabolic and physiological features with humans, which have long made them appealing animal models for infectious disease research^2^. Unlike immunocompetent rodent models, ferrets offer the advantage of being susceptible to most wild type filoviruses, particularly orthoebolaviruses^1^, eliminating the need for host adaptation. Additionally, ferrets recapitulate many features of human filovirus disease (FVD), including high levels of virus replication and a dysregulated immune response. Ferrets have been shown to be highly susceptible to several orthoebolaviruses, namely Ebola virus (EBOV)^3–7^, Sudan virus (SUDV)^3,8^, Bundibugyo virus (BDBV)^3,4^, and Reston virus (RESTV)^9^, all of which are uniformly lethal among infected animals. Taï Forest virus (TAFV) is non-lethal in ferrets, resulting in asymptomatic infection^10^. Unlike most orthoebolaviruses, infection with orthomarburgviruses, namely Marburg virus (MARV) and Ravn virus (RAVV), does not cause disease in ferrets regardless of challenge variant, dose and route^7,11,12^.

The fact that ferrets are uniformly susceptible to infection with most orthoebolaviruses but not orthomarburgviruses suggests that these viruses differ fundamentally in their pathogenic mechanisms. Recent evidence from our lab demonstrated that the lack of disease among MARV-inoculated ferrets is likely not the result of a block in GP-mediated entry^7^. Indeed, ferrets were found to be uniformly susceptible to infection with a chimeric EBOV expressing MARV GP in place of EBOV GP, ultimately suggesting that the block lies downstream of viral entry^7^.

In immunocompetent rodents, including mice and guinea pigs, wild type EBOV and MARV are non-lethal^13–17^. To overcome this, several groups have established uniformly lethal host-adapted viruses, most of which were developed via the serial passaging of these viruses within their respective rodent hosts. Among these adapted viruses are multiple variants of mouse-adapted (MA)-EBOV^18,19^, MA-MARV^20,21^, guinea-pig adapted (GPA)-EBOV^22,23^ and GPA-MARV^24,25^.

Here, we set out to investigate the pathogenicity of GPA-MARV and MA-MARV in ferrets in an attempt to establish a uniformly lethal model. Our results demonstrated that GPA-MARV was uniformly lethal among infected ferrets, with all animals succumbing to disease 10 days post-infection (dpi). In contrast, MA-MARV was only partially lethal, with 33% of animals reaching the humane endpoint at 8-9 dpi. All lethally infected animals demonstrated clinical signs of disease characteristic of FVD in ferrets, including high viremia, disturbances in hematological parameters, and a dysregulated immune response, notably a proinflammatory cytokine response. Moreover, some animals infected with GPA-MARV, but none of those infected with MA-MARV, developed a petechial rash. Interestingly, we identified several genome mutations in GPA-MARV that represented critical mutations present among previously established rodent-adapted MARV variants. This represents the first report and characterization of a lethal orthomarburgvirus ferret model, ultimately making this ferret model appealing for both countermeasure and pathogenic evaluations. This described model is particularly relevant at present as it can be used to screen candidate countermeasures that are desperately needed to combat the ongoing MARV outbreak in Rwanda^26^.

## Results

### GPA-MARV is uniformly lethal in ferrets

**Fig. 1:**
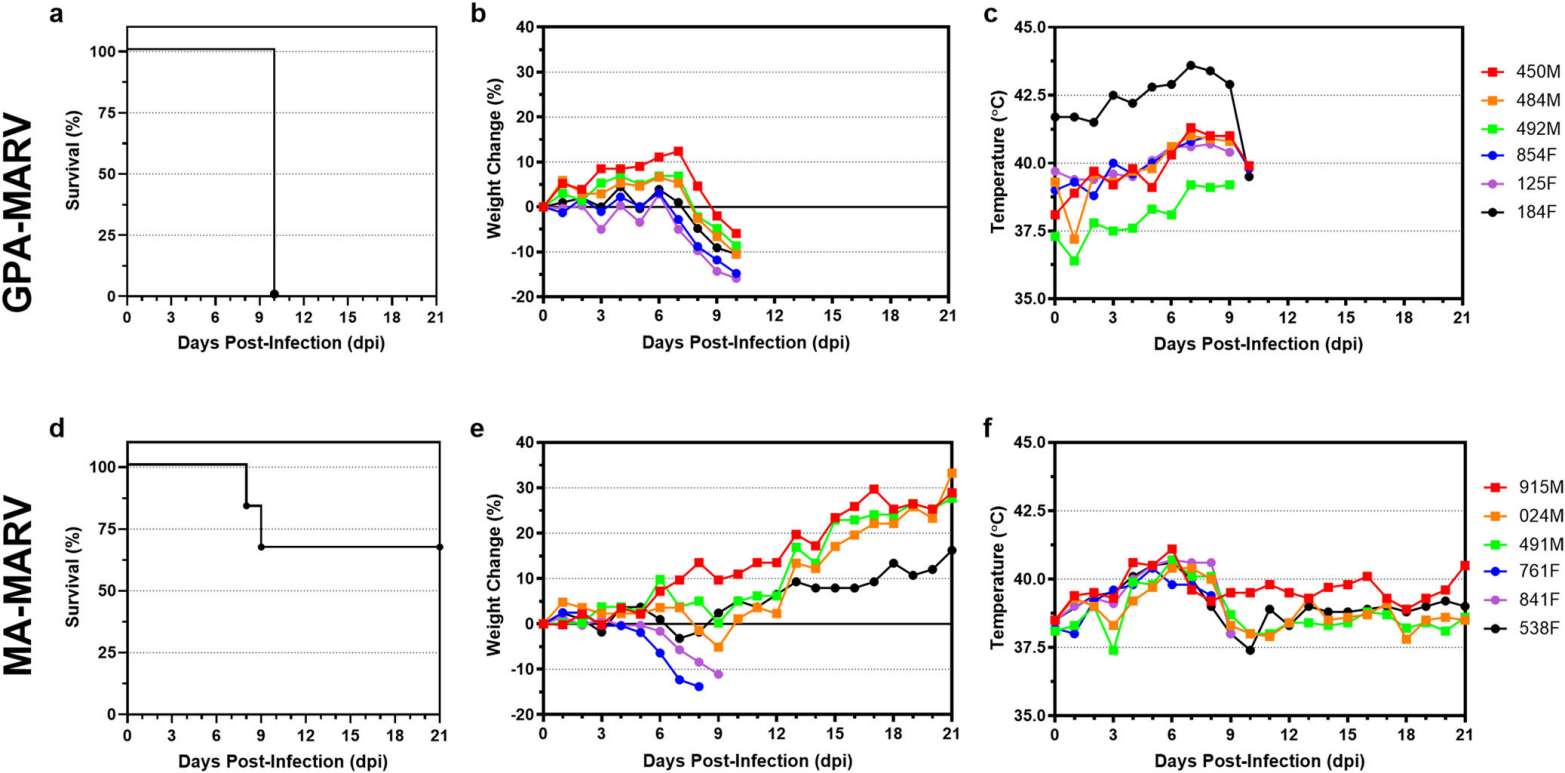
Clinical parameters of ferrets inoculated with rodent-adapted Marburg viruses. Clinical parameters of ferrets (*n* = 6 per group) inoculated with GPA-MARV (**a**–**c**) or MA-MARV (**d–f**). Kaplan-Meier survival curves (**a**, **d**), percent weight change (**b**, **e)**, microchip scan temperature (**c**, **f**). Data from each animal are depicted as dots with individual animal IDs and sex (Female, F and Male, M) indicated in the key.

**Fig. 2:**
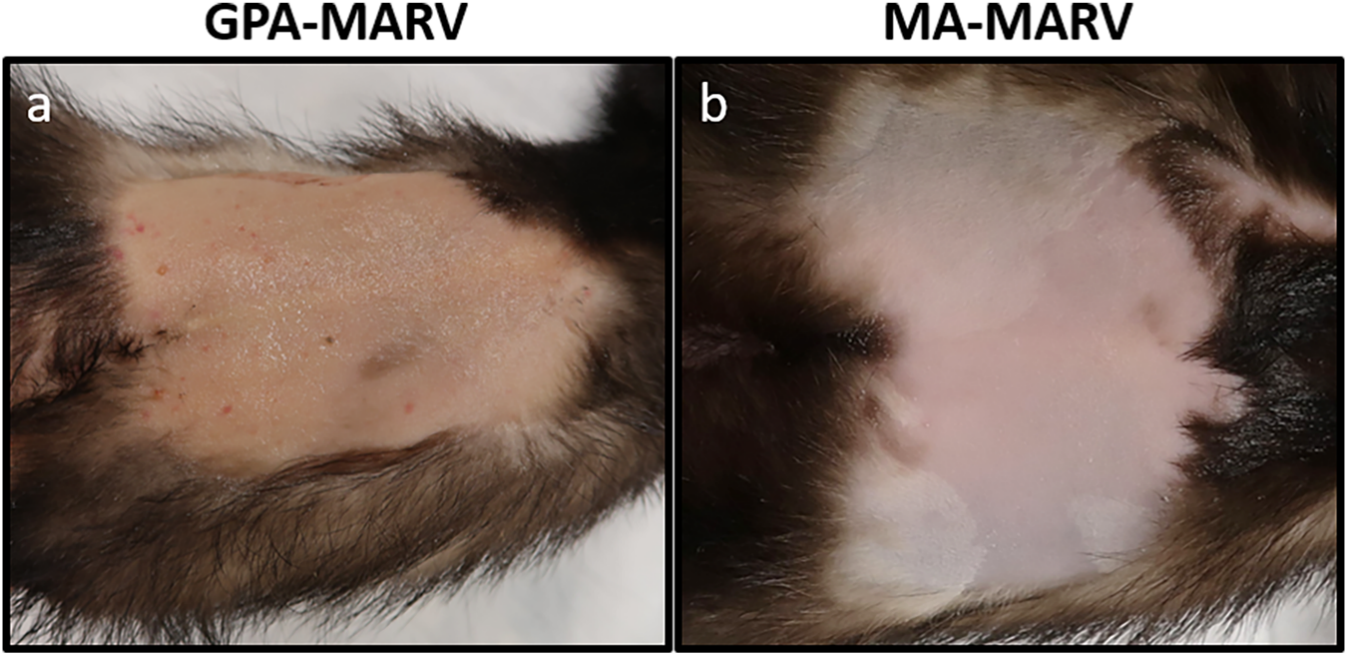
Rash observed among ferrets infected with rodent-adapted Marburg viruses. Representative images of rashes observed at the terminal timepoint. Petechial rash among GPA-MARV-challenged ferrets (**a**) and no rash among MA-MARV-challenged ferrets (**b**).

To determine whether GPA-MARV or MA-MARV caused disease in ferrets, groups of six male and female ferrets were inoculated with 1000 TCID_50_ of each virus. A third group of ferrets was inoculated with GPA-EBOV as a control. Remarkably, GPA-MARV resulted in uniform lethality, with all animals succumbing to disease at 10 dpi (Fig. 1a). These animals began manifesting clinical signs of FVD at 7-8 dpi, which coincided with weight loss and an increase in body temperature (Fig. 1b, c). Beyond 8 dpi, disease progressed rapidly, with the most notable signs of disease being significantly decreased activity, inappetence, and a hunched posture with ruffled fur. Two of the six ferrets challenged with GPA-MARV demonstrated a petechial rash observable roughly 24–48 h prior to the time of euthanasia (Fig. 2a). Additionally, a few animals showed signs of hemorrhage other than a rash, including blood in the stool as well as some orifices, including nose and eyes.

MA-MARV resulted in partial lethality among infected ferrets, with two of the six animals (33%) euthanized on 8 and 9 dpi (Fig. 1d). These two animals exhibited signs of disease that appeared less severe than those observed for GPA-MARV, with weight loss progressing gradually from 3 dpi until euthanasia and temperatures staying within normal range or mildly elevated (Fig. 1e, f). Neither of the animals that succumbed developed a rash at any point (Fig. 2b). The other four MA-MARV-challenged animals did not exhibit any signs of disease (Fig. 1d–f).

By comparison, all ferrets challenged with GPA-EBOV succumbed to disease by 5 dpi (Supplementary Fig. 1a). These animals manifested clinical signs of disease that closely paralleled what has been observed previously for EBOV^4,7^ (Supplementary Fig. 1a–c). In contrast to GPA-MARV-challenged ferrets, among which only two animals developed a petechial rash, all GPA-EBOV-challenged ferrets exhibited a maculopapular rash (Supplementary Fig. 1d). Notably, the rash observed among GPA-MARV-challenged animals differed from that observed among GPA-EBOV-challenged animals, with the latter displaying a maculopapular rash that blanched upon applying pressure.

### GPA-MARV replicates to high levels in ferrets

**Fig. 3:**
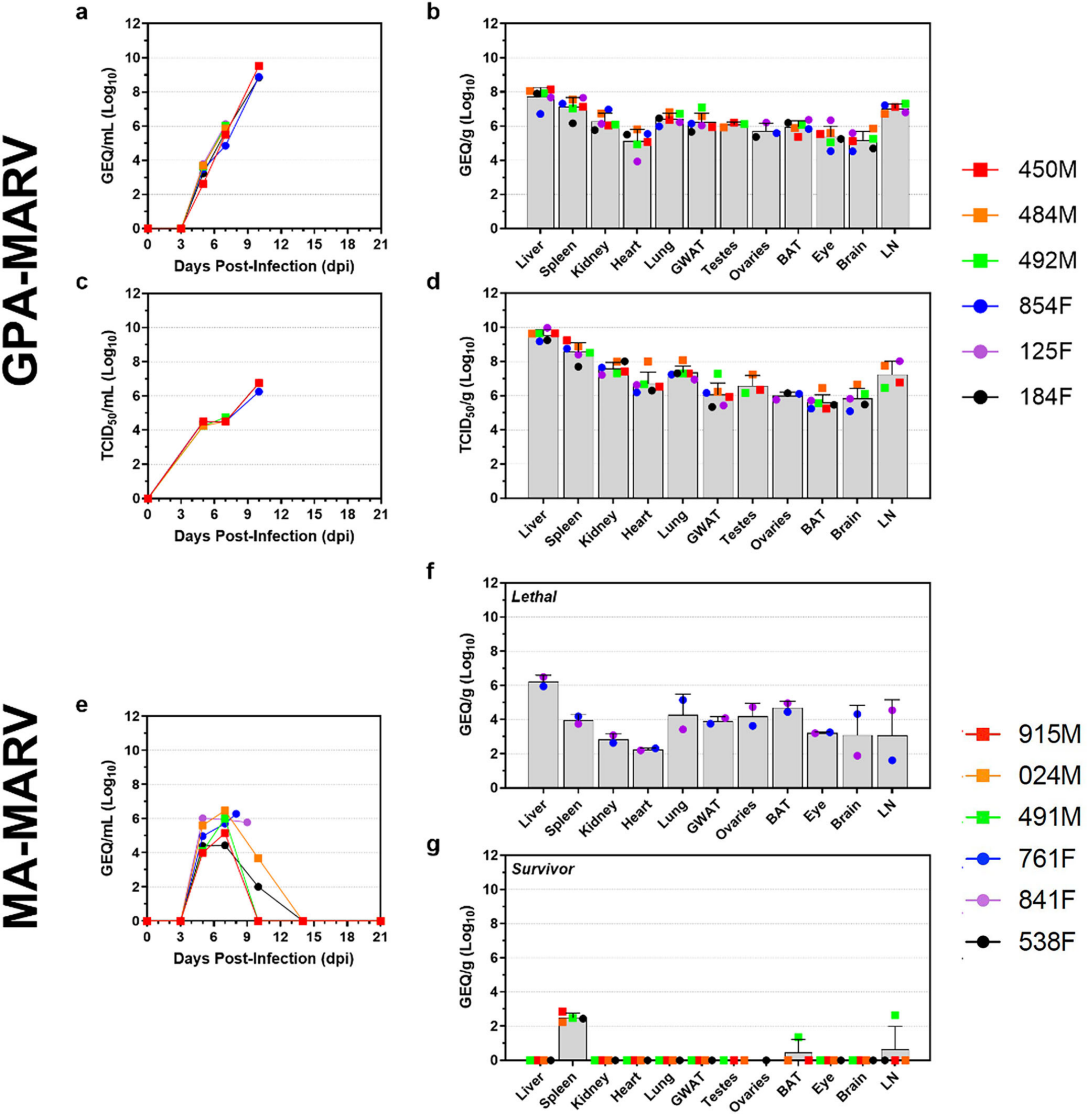
Viral load in blood and tissues of ferrets infected with rodent-adapted Marburg viruses. Whole blood (**a**, **c**, **e**) was collected from each animal at 3, 5, 7, 10, 14 and 21 days post-infection (dpi) and at the time of euthanasia, while tissues (liver, spleen, kidney, heart, lung, gonadal white adipose tissue (GWAT), testes, ovaries, brown adipose tissue (BAT), eye, brain, and lymph node (LN)) were collected upon necropsy to evaluate viral loads among animals challenged with GPA-MARV (**a–d**) and MA-MARV (**e–g**). Virus RNA was quantified by RT-qPCR (**a**, **b**, **e**, **f**, **g**), and infectious virus was quantified by TCID_50_ (**c**, **d**). Results are depicted in genome equivalents per mL (GEQ/mL) for each animal (indicated by a square for males and a circle for females) or mean tissue culture infectious dose per mL (TCID_50_/mL) or per g (TCID_50_/g) depicted as histograms, with data from each animal depicted as dots. Individual animal IDs and sex (Female, F and Male, M) are indicated in the key. Note: virus isolation was only performed on samples positive by RT-qPCR with a CT cutoff of ≤34.

All animals infected with GPA-MARV were viremic on 5 dpi, with low levels of viral RNA detectable in the blood (mean 3.40 Log_10_ GEQ/mL). Levels of viral RNA increased moderately by 7 dpi with a mean terminal level of 9.07 Log_10_ GEQ/mL (Fig. 3a). Oral, rectal and nasal swabs were weakly positive from 5-7 dpi, and mean viral RNA levels increased to 5.97, 7.78, 6.38 Log_10_ GEQ/mL, respectively, at the time of euthanasia (Supplementary Fig. 2a–c). Tissues were uniformly positive for viral RNA at the time of euthanasia, with liver, spleen and lymph nodes having the highest mean levels of viral RNA, at 7.73, 7.14 and 7.03 Log_10_ GEQ/g, respectively (Fig. 3b). Levels of infectious virus in the blood and tissues paralleled the levels of viral RNA (Fig. 3c, d), while the swabs were weakly positive for infectious virus (data not shown).

In contrast, all MA-MARV-challenged animals were viremic at 5 dpi, with mean levels of viral RNA in the blood of 4.85 Log_10_ GEQ/mL (Fig. 3e). Levels of viral RNA in the blood increased slightly at 7 dpi, with mean levels of 5.61 Log_10_ GEQ/mL, and began to decrease thereafter in all surviving animals (Fig. 3e). The two animals that succumbed to disease had mean viral RNA levels of 6.03 Log_10_ GEQ/mL in the blood at the time of euthanasia (Fig. 3e). Swabs were mostly negative throughout the duration of the study, with the most positives being found in oral swabs at 7 dpi (Supplementary Fig. 2g–i). The two animals that succumbed to disease at 8 and 9 dpi were uniformly positive for viral RNA across all tested tissues; however, levels were considerably lower than those observed for GPA-MARV (Fig. 3f). Among the surviving animals at 21 dpi, the spleen was weakly positive for viral RNA, while a single animal showed low levels of viral RNA in the BAT and LN (Fig. 3g).

Viral replication kinetics for GPA-EBOV-challenged animals closely paralleled what has been observed previously^7^, with mean peak levels of 10.95 Log_10_ GEQ/mL in the blood at time of euthanasia on 4 dpi (Supplementary Fig. 3a). Likewise, all tissues and swabs were uniformly positive for viral RNA and infectious virus at time of euthanasia (Supplementary Fig. 2d–f and Supplementary Fig. 3b). Overall, levels of viral RNA at the time of euthanasia were ~2 Log_10_ GEQ/mL greater among GPA-EBOV-challenged animals compared to GPA-MARV-challenged animals.

### GPA-MARV causes deviations in hematological parameters

**Fig. 4:**
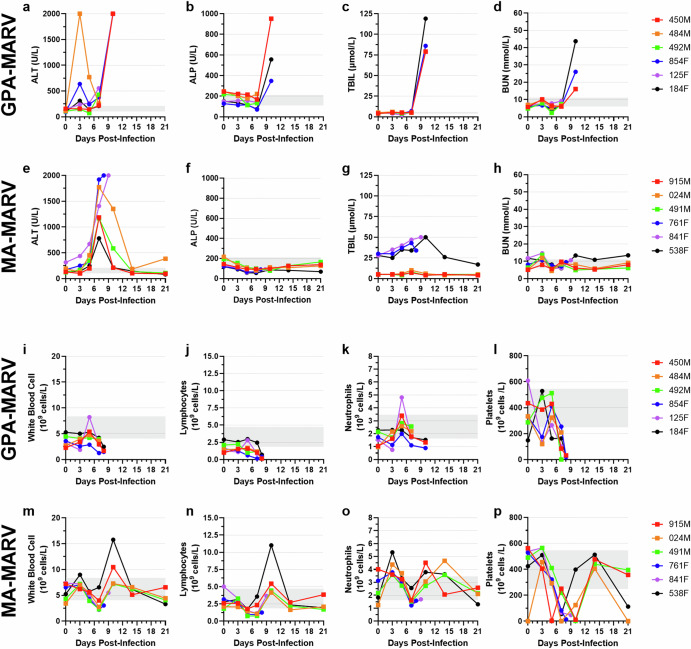
Select biochemistries and blood cell counts in ferrets infected with rodent-adapted Marburg viruses. Whole blood was collected from each animal on days 0, 3, 5, 7, 10, 14 and 21 days post-infection (dpi) as well as at the time of euthanasia to evaluate serum biochemistry (**a–h**) and blood cell counts (**i–p**). Biochemistry analytes measured are as follows: alanine aminotransferase [ALT]; alkaline phosphatase [ALP]; total bilirubin [TBIL], and blood urea nitrogen [BUN]. Complete blood count analytes measured are as follows: white blood cells [WBC]; lymphocytes [LYM]; neutrophils [NEU]; platelets [PLT]. Individual animal IDs and sex (Female, F and Male, M) are indicated in the key. Gray shaded regions represent the mean baseline values ± 2 standard deviations, calculated from historical pre-challenge serum samples. Note: Partial timepoint sampling was performed for animals that succumbed to disease or met humane endpoint criteria prior to the scheduled end of the study. This includes all animals in the GPA-MARV group, as well as animals 761 F and 841 F in the MA-MARV group.

GPA-MARV-challenged animals demonstrated deviations in blood biochemistry characteristic of FVD. ALT levels were elevated in most animals as early as 3 dpi, reaching the upper limit of quantification (2000 U/L) in three animals (450 M, 854 F and 184 F) at the time of euthanasia (Fig. 4a). Because the remaining three animals (484 M, 492 M and 125 F) were found dead on 10 dpi, no hematology data were available at this timepoint. ALP, TBIL and BUN levels were relatively stable throughout 7 dpi but were elevated at 10 dpi for 450 M, 854 F and 184 F (Fig. 4b–d). The remaining analytes, namely, ALB, AMY, Ca^2+^, PHOS, CRE, GLU, Na^+^, K^+^, TP and GLOB, were mostly unremarkable, except AMY, PHOS and K^+,^ which was elevated for a single animal (184 F) at the time of euthanasia (Supplementary Fig. 4).

With respect to MA-MARV, ALT levels for the non-surviving animals (761 F and 841 F) were elevated as early as 3 dpi and continued to increase until reaching the upper limit of quantification (ULOQ) at the time of euthanasia (Fig. 4e). ALT levels for the surviving animals peaked at 7 dpi, after which they gradually decreased (Fig. 4e). Levels of ALP and BUN remained mostly within the normal range throughout 21 dpi (Fig. 4f, h). Interestingly, TBIL levels were within normal range for males but elevated for females throughout the study (Fig. 4g). Also of note, GLOB levels increased for all animals as early as 3 dpi and peaked at 7 dpi before returning to baseline at 21 dpi (Supplementary Fig. 4).

In GPA-EBOV-challenged animals, ALT, ALP, TBIL and BUN were elevated at the time of euthanasia, consistent with what is seen among ferrets challenged with EBOV^4^ (Supplementary Fig. 4**)**. Unlike GPA-MARV, levels of PHOS, CRE, K^+^ and GLOB were also elevated among GPA-EBOV-challenged animals at euthanasia (Supplementary Fig. 4).

GPA-MARV and MA-MARV challenged animals all demonstrated lymphopenia and thrombocytopenia at the time of euthanasia (Fig. 4j, l, n, p). In the case of MA-MARV platelets reached their lowest levels at 7 dpi and gradually returned to normal levels among survivors by the study end (Fig. 4p). No discernable trend among white blood cell levels could be observed for GPA-MARV challenged animals (Fig. 4i). In contrast, MA-MARV-challenged animals 915 M and 538 F demonstrated elevated white blood cell levels at 10 dpi, which returned to baseline levels by study end (Fig. 4m). Interestingly, neutrophils were elevated at 5 dpi among GPA-MARV-challenged animals and returned to baseline prior to the time of euthanasia (Fig. 4k). In contrast, MA-MARV-challenged animals demonstrated elevated levels of neutrophils at 3 dpi, which decreased at 7 dpi followed by and increase thereafter among surviving animals (Fig. 4o). Lastly, GPA-EBOV-challenged animals demonstrated trends consistent with what has been shown previously among ferrets infected with EBOV (Supplementary Fig. 5).

### Infected ferrets demonstrate a dysregulated immune response

**Fig. 5:**
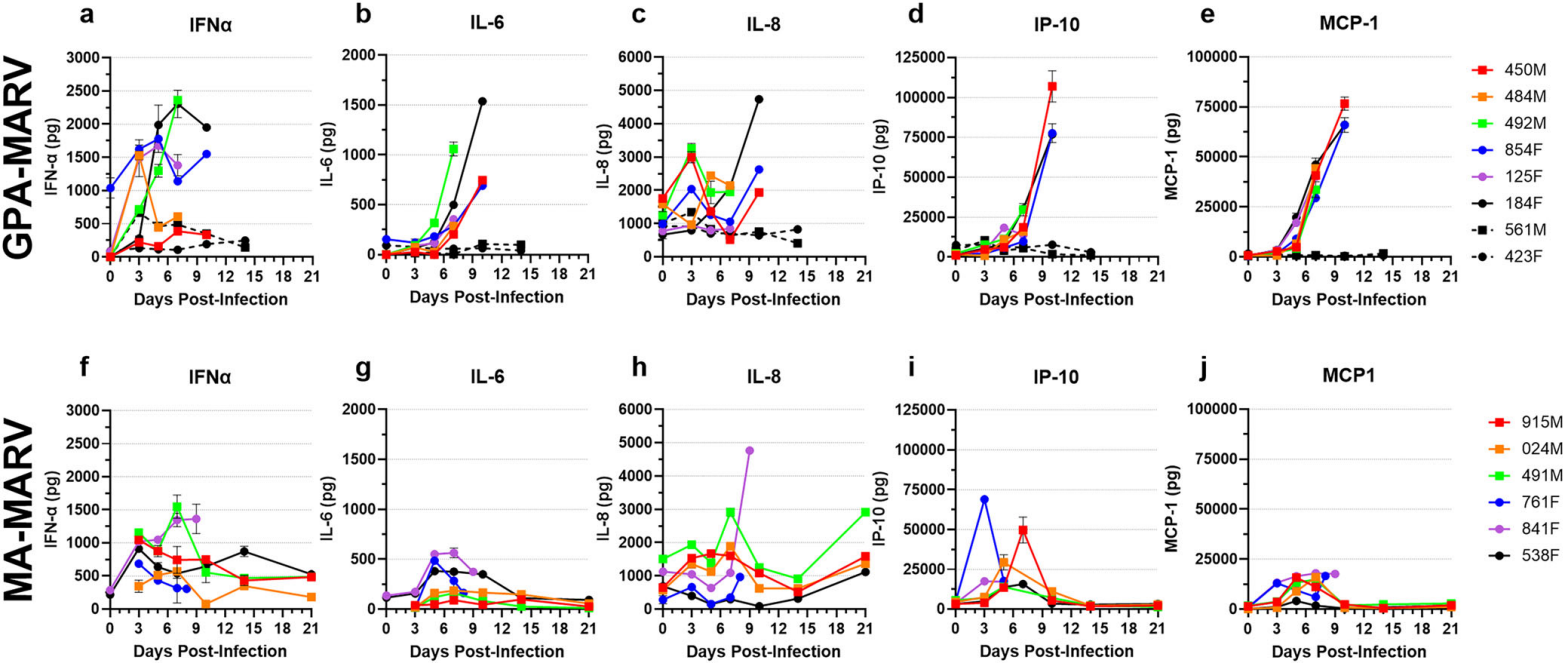
The cytokine/chemokine response in ferrets challenged with rodent-adapted Marburg viruses. Serum was collected from each animal challenged with GPA-MARV (**a–e**) or MA-MARV (**f–j**) at 0, 3, 5, 7, 10, 14 and 21 days post-infection (dpi) as well as at the time of euthanasia to evaluate cytokine/chemokine profiles using a 12-plex ferret Luminex assay. Analytes measured are as follows: Interferon-alpha [IFNα]; interleukin-6 [IL-6]; interleukin-8 [IL-8]; Interferon gamma-induced protein 10 [IP-10]; monocyte chemoattractant protein-1 [MCP-1]. Individual animal IDs and sex (Female, F and Male, M) are indicated in the key. Animals 561 M and 423D, denoted by hashed lines, reflect historical control sera from ferrets challenged with wild type and MARV (*n* = 2) from a previously published study^7^.

Ferret serum samples were subjected to a 12-plex ferret cytokine panel to investigate changes in the cytokine profile that occurred following infection. In GPA-MARV-challenged animals, the proinflammatory cytokines IFN-α, IL-6, IL-8, IP-10 and MCP-1 all increased following infection, with levels peaking in most animals at the time of euthanasia (Fig. 5a–e). Notably, these cytokines were all substantially elevated relative to historical control animals challenged with wild type MARV (Fig. 5a–e). Interestingly, animal 492 M showed consistently elevated levels of TNF-α, IL-12P70, IL-12p40, IL-17 and IL-2 compared to all other animals challenged with GPA-MARV (Fig. 5 and Supplementary Fig. 7). Cytokine levels for TNF-α, IL-12P70, IL-12p40, IL-17, IL-2, IL-4 and MIP-1β showed considerable fluctuation throughout the course of disease with no discernable trends (Fig. 5 and Supplementary Fig. 7).

Trends observed for MA-MARV-challenged animals deviated from those observed among GPA-MARV-challenged animals. The dramatically elevated levels of IFN-α, IL-6, and MCP-1 observed among GPA-MARV-challenged animals were absent among MA-MARV-challenged animals (Fig. 5f, g, j). Nonetheless, slight increases were seen among these analytes around 5-7 dpi; however, these levels normalized by 10 dpi in the survivors (Fig. 5f, g, j). Interestingly, levels of IL-8 increased in all animals around 7 dpi, coinciding with disease onset and clinical scoring among the two animals that succumbed. Among the surviving animals, IL-8 levels decreased at 14 dpi only to increase again at the time of euthanasia (Fig. 5h). For unknown reasons, levels of IL-17 and IL-4 were elevated throughout the study for 841 F and 538 F compared to animals of the same challenge group (Supplementary Fig. 7). The levels of the remaining analytes, TNF-α, IP-10, IL-12p70, IL-12p40, IL-2, MCP-1 and MIP-1β, fluctuated, with peaks observed around 7 dpi followed by decreases thereafter (Fig. 5 and Supplementary Fig. 7).

In general, trends observed among GPA-EBOV-challenged animals were similar to those observed among GPA-MARV-challenged animals, and they closely paralleled those observed among historical control animals challenged with wild type EBOV (Fig. 5 and Supplementary Fig. 6). In particular, IL-6 and IL-8 levels in GPA-EBOV-challenged animals at the terminal timepoint far exceeded those observed at the terminal timepoints in GPA-MARV-challenged animals (Supplementary Fig. 6). IP-10 was also elevated at the terminal timepoint among GPA-EBOV-challenged animals, although to levels lower than those observed for GPA-MARV (Supplementary Fig. 6). Levels of IFNα, TNFα, IL-12p70, IL-17, IL-2, IL-4 and MIP-1β showed fluctuations throughout the disease course with no discernable trends (Supplementary Fig. 6).

### GPA-MARV acquired several mutations following challenge

To track changes in the virus genome throughout the course of infection, whole blood collected at 3, 5, and 7 dpi and at the time of euthanasia, along with tissues, were subjected to NGS. In total, 28 mutations were identified within GPA-MARV, of which eight were located within coding regions (Table 1 and Supplementary Figs. 8, 9). All of these mutations were found in each GPA-MARV-challenged animal in all tissues and blood samples. All mutations within the coding regions—except a single mutation in VP35 were non-synonymous, including one in NP (F287L), two in VP35 (L30S and Y34H), three in VP40 (K56N, G79S and D184N) and one in VP24 (I66V). Each of these non-synonymous mutations was present within the virus challenge stock at frequencies below 25% except K56N in VP40 which was present at a frequency of 34.01% (Table 1); however, all reached the consensus threshold frequency of ≥50%, with the majority present at a frequency of ≥75%. The remaining 20 mutations were silent and located either within UTRs or intergenic regions (Table 1). In addition to these 28 mutations, several additional mutations were present; however, most were unique to a single animal or tissue and demonstrated no discernible trends of interest. Unfortunately, given the low viral load among MA-MARV-challenged animals NGS was not feasible.

**Table 1:** Summary of mutations present in blood and tissues of GPA-MARV challenged ferrets

Location	Nucleotide Mutation^a^	Amino Acid Mutation	Mutation Frequency in Challenge Stock (%)
NP	T1562C	F287L	14.36
VP35	A2931T	-	
	T3033C	L30S	16.48
	T3044C	Y34H	19.51
IR VP35:VP40	T4114C	-	
5’ UTR VP40	T4116C	-	
	T4121C	-	
	T4122C	-	
	T4129C	-	
	T4130C	-	
	T4148C	-	
	T4153C	-	
	T4154C	-	
	T4157C	-	
	T4171C	-	
	T4182C	-	
	T4201C	-	
	T4236C	-	
	T4273C	-	
	T4274C	-	
	T4324C	-	
	T4325C	-	
	T4326C	-	
VP40	A4735T	K56N	34.01
	G4802A	G79S	7.40
	G5117A	D184N	11.04
3’ UTR GP	G8309A	-	
VP24	A10402G	I66V	21.30

*IR* intergenic region, *UTR* untranslated region.

^a^Mutations reported are those that reach the consensus threshold of ≥50% and were conserved across each GPA-MARV-challenged animal and present among all tissues harvested at the time of necropsy and blood samples for all timepoints. Abbreviations: IR (intergenic region), UTR (untranslated region).

## Discussion

The fact that most orthoebolaviruses cause uniformly lethal disease in ferrets, while orthomarburgviruses do not, is surprising given that many of these viruses cause severe, often fatal, disease in humans and ΝΗPs that is at least superficially similar. Several rodent-adapted viruses, including GPA-MARV and MA-MARV, have been established in recent years and cause lethal disease in rodents, which, akin to ferrets, are refractory to infection with wild type virus. Given that many of the mutations present within these adapted viruses have been found to contribute to virus virulence in rodents, we wondered whether they might also contribute to pathogenicity in ferrets.

Remarkably, challenge with GPA-MARV resulted in uniform lethality among ferrets, whereas challenge with MA-MARV resulted in partial lethality. Interestingly, the onset of clinical signs of disease and time of euthanasia among lethally infected MA-MARV ferrets occurred roughly one day before GPA-MARV challenged animals. Furthermore, lethally infected MA-MARV animals manifested clinical signs of disease that closely paralleled what was observed among GPA-MARV challenged animals, whereas MA-MARV survivors did not manifest any signs of disease. Surprisingly, some GPA-MARV- challenged animals—but none of the MA-MARV-challenged animals—developed a petechial rash characteristic of what is observed in NHPs. Moreover, the onset of viremia for both challenged groups occurred at 5 dpi; however, levels of viral RNA within the blood were ~1–2 Log_10_ higher in MA-MARV-challenged animals compared to GPA-MARV-challenged animals. This could suggest that the adaptations present within MA-MARV provide a slight advantage over GPA-MARV regarding viral replication, at least during the early stages of infection. Despite this early advantage, terminal levels of viral RNA in the blood were ~3–4 Log_10_ greater among GPA-MARV-challenged animals compared to lethally infected MA-MARV-animals. Further, levels of viral RNA in tissues were roughly 2 log_10_ GEQ/mL lower for animals lethally infected with MA-MARV compared to GPA-MARV. The lower levels of viremia among MA-MARV-challenged animals correlate with milder disease.

GPA-MARV-challenged animals demonstrated elevated levels of ALT, ALP, TBIL and BUN, indicating liver and kidney dysfunction consistent with observations in NHPs^27^. In contrast, ALP, TBIL, and BUN levels did not change throughout the study among animals challenged with MA-MARV, indicative of a mild disease. Furthermore, levels of ALT spiked around 7-8 dpi for MA-MARV-challenged animals and normalized shortly after among survivors consistent with the resolution of disease. Additionally, GPA-MARV-challenged animals exhibited thrombocytopenia, a previously undescribed phenomenon among EBOV-challenged animals. This could also be observed among MA-MARV-challenged animals; however, PLT levels normalized among surviving animals. These low PLT levels represent a hallmark feature of Marburg virus disease (MVD) and are frequently seen in NHPs^27^, but this represents the first instance seen in ferrets challenged with orthomarburgviruses.

A strong cytokine response was observed among GPA-MARV-challenged animals but not among MA-MARV-challenged animals. More specifically, GPA-MARV-challenged animals demonstrated elevated levels of proinflammatory cytokines IL-6, IL-8, and IP-10. The elevated levels observed for IFN-α, IL-6, and MCP-1 among GPA-MARV-challenged animals were absent among MA-MARV-challenged animals. Slight increases were seen among these analytes around 5-7 dpi; however, these levels normalized shortly after at 10 dpi. These cytokine data observed among GPA-MARV- and GPA-EBOV-challenged animals are consistent with what is typically observed among humans and NHPs^28^. In contrast, the minimal cytokine response observed among MA-MARV-challenged animals correlates with the lack of lethal disease among most animals. Interestingly, no major difference could be observed among the cytokine profiles of ferrets that survived MA-MARV challenged and those that had lethal outcomes.

Sequencing revealed 28 mutations present in the virus genomes isolated from both the tissues and blood of all ferrets challenged with GPA-MARV, of which eight were located within coding regions. These mutations within coding regions were all present in the virus challenge stock at a frequency ≤ 25%. Among these were two non-synonymous mutations, K56N in VP40 and I66V in VP24, that represented reversions to the sequence of wild type MARV Angola (GenBank: DQ447653.1). Four of the non-synonymous mutations, namely L30S and Y34H in VP35 and G79S and D184N in VP40, have previously been identified in other mouse-adapted orthomarburgviruses: L30S and Y34H are present within MA-MARV variant Angola (MA-MARV/Ang)^20^; G79S is present within both MA-MARV variant Ci67 (MA-MARV/Ci67)^29^ and MA-MARV/Ang^20^; and D184N is present within MA-MARV/Ci67, MA-RAVV^29^ and MA-MARV/Ang^20^. While the function of many of these mutations has yet to be elucidated, the fact that they reach ≥50% frequency post-challenge and are present in multiple mouse-adapted orthomarburgviruses suggests an important role of these mutations towards viral pathogenesis in vivo. Furthermore, it is reasonable to speculate that the mutations within VP35 and VP40 contribute to the virus’s ability to subvert and evade the host’s innate immune response, potentially contributing to the lethality of GPA-MARV within ferrets. Particularly, both VP35 and VP40 have been shown to function as antagonists of the innate immune response critical for inhibiting different stages of the IFN response^30^. Indeed, the G79S mutation has been found to enhance VP40 ability to function as an antagonist of type I IFN signaling. In contrast, D184N has been shown to contribute to VP40 role as a viral matrix protein and has no effect on the ability of VP40 to inhibit the IFN response in guinea pig or human cells^31,32^. Lastly, the non-synonymous mutation F287L in NP has not previously been identified and may contribute to the pathogenesis of GPA-MARV in some unknown manner. With the speculation that the mutations present within GPA-MARV contribute to the virus’s lethality within ferrets, future investigations should focus on elucidating the molecular determinants of disease using reverse genetics.

Compared to the GPA-EBOV-challenged animals, onset of disease and viremia was delayed among ferrets challenged with rodent-adapted Marburg viruses by approximately two days, suggesting orthoebolaviruses may be more efficient at causing disease within ferrets. These differences in levels of viral RNA could possibly be correlated with the reduced efficiency of GP-mediated entry and attenuated replication kinetics of orthomarburgviruses observed in vitro within ferret cell lines^7^. Indeed, studies from our lab recently demonstrated the efficiency by which MARV GP mediates entry into ferret cells is poor compared to that of EBOV, potentially contributing to the lack of disease among MARV-challenged ferrets. Despite this, levels of viral RNA among tissues at terminal timepoint were roughly similar among GPA-MARV and GPA-EBOV but approximately 2 log_10_ GEQ/mL lower for animals lethally infected with MA-MARV. Nonetheless, clinical manifestations of disease, high viremia, disturbances in hematological parameters, and dysregulated immune response among GPA-EBOV challenged animals closely recapitulate what has been observed previously among EBOV challenged ferrets^4,7^.

This study represents the first description of a lethal ferret model for MARV. The described GPA-MARV ferret model draws many parallels to the disease features observed among NHPs, including high viremia, disturbances in hematological parameters, and a dysregulated immune response characterized by a proinflammatory cytokine response^27,33^. Moreover, the extended disease window of 10 days following challenge with GPA-MARV compared to only 4 days for EBOV may make this ferret model appealing for countermeasure evaluation, particularly for small molecules and drugs. However, one major drawback to this model is that an adapted virus is required to achieve uniform lethality, which is not the case for EBOV. Nonetheless, the described model represents a significant advancement to filovirus animal modeling and may prove pivotal as a precursor model prior to investigations in NHPs. Moreover, the described model is extremely relevant at present as it could be used for the screening of candidate countermeasures, which are desperately needed to help combat the current MARV outbreak in Rwanda, which at present is third largest in history^26^.

## Methods

### Viruses

GPA-EBOV (variant Mayinga, Pathoplexus Accession# PP_002YX4W.1), GPA-MARV (variant Angola, GenBank: MF939097.1), and MA-MARV (variant Angola) were established as described previously^20,22,25^. Next-generation sequencing (NGS) identified two mutations in MA-MARV relative to GenBank Accession No. KM261523.1: an amino acid mutation D422N in the GP coding sequence and a nucleotide mutation T8378C in GP 5’-untranslated region (UTR). All virus stocks used for animal infections were negative for mycoplasma contamination.

### Animals

Male (*n* = 9) and female (*n* = 9) ferrets (*Mustela putorius furo)* ~ 5–6 months old and weighing 0.65–0.7 kg were purchased from Marshall BioResources (New York, USA) and implanted with an IPTT-300 temperature and ID transponder (BioMedic Data Systems Inc., USA). Following acclimatization, ferrets were randomly assigned into groups containing equal numbers of males and females and inoculated with either GPA-EBOV (*n* = 6), GPA-MARV (*n* = 6) or MA-MARV (*n* = 6). Virus inoculums were prepared in Dulbecco’s Modified Eagle Medium (DMEM) and administered bilaterally at two sites (250 μL/rear quadricep) via the intramuscular (IM) route. The target inoculation dose was 1000 TCID_50_, and the inoculums were back titrated to doses of 1581, 2.81 × 104 and 3749 TCID_50_ for GPA-MARV, MA-MARV and GPA-EBOV, respectively. Following inoculation, animals were monitored daily for clinical signs of disease, including changes in body weight, temperature, physical activity, and food/water intake. Animals that reached the clinical scoring criteria for humane endpoint were euthanized. Whole blood (EDTA), plasma (lithium heparin) and serum were collected pre-infection (Pre) as well as at 3, 5, 7, 10, 14 and 21 dpi and at the time of euthanasia for downstream analysis. Oral, rectal and nasal swabs were collected using cotton swabs and placed into 750 μL unsupplemented DMEM at the aforementioned time points. Tissues, including liver, spleen, kidney, heart, testes, ovaries, gonadal white adipose (GWAT), brown adipose (BAT), eyes and brain, were harvested at the time of necropsy from all animals for downstream analysis.

### Plasma biochemistry and complete blood counts

Plasma biochemistries were evaluated using the VetScan VS2 blood analyzer (Abaxis, USA), according to the manufacturer’s instructions, using whole blood collected in lithium heparin tubes (BD, USA). Complete blood counts (CBC) were evaluated using the VetScan HM5 hematology system (Abaxis, USA), using whole blood collected in EDTA tubes (BD, USA).

### Quantification of viral RNA by RT-qPCR

Total RNA was extracted from whole blood, as well as oral, rectal and nasal swabs, using the QIamp viral RNA minikit (Qiagen, USA) or the KingFisher Apex using the Viral/Pathogen II Nucleic Acid Isolation Kit (Applied Biosystems, USA). Extractions done using the QIamp viral RNA minikit were performed according to the manufacturer’s instructions, with the sole modification being that a second AW1 wash was performed for whole blood. Total RNA was extracted from 30 mg homogenized tissues using the KingFisher Apex using the same kit described above, except that RNA was eluted in 50 μL elution buffer. Levels of viral RNA were quantified by RT-qPCR using the TaqPath™ 1-Step Multiplex Master Mix (Applied Biosystems, USA) according to the manufacturer’s protocol using primers and probes targeting either EBOV L or MARV L (Supplementary Table 1). RT-qPCR reactions were done using a Quantstudio 5 Real-time PCR system (Applied Biosystems, USA) with the following thermocycling conditions: 25 °C for 2 min, 53 °C for 10 min and 95 °C for 2 min, followed by 40 cycles of 95 °C for 3 s, 60 °C for 30 s.

### Quantification of infectious virus by TCID_50_

Vero E6 cells were seeded in 96-well plates at a cell density of 2.0 × 104 cells/well to achieve a confluency of 90–95% after 24 h. The following day, the medium was aspirated and replaced with DMEM supplemented with 2% heat-inactivated fetal bovine serum (HI-FBS), 1% L-glutamine and 1% penicillin/streptomycin. Tissues were homogenized in 1000 μL DMEM containing a 5-mm steel bead using the Bead Ruptor Elite (Omni International, USA) and clarified by centrifugation. Whole blood, swabs or clarified tissue homogenate (40 μL) were diluted in 360 μL of DMEM supplemented with 2% HI-FBS, 1% L-glutamine and 1% penicillin/streptomycin and serially diluted 10-fold (10^−1^–10^−8^). Diluted samples (100 μL) were added to the Vero E6 cells and incubated at 37 °C and 5% CO_2_ for 14 days, at which time cytopathic effects (CPE) were observed. The median tissue culture infectious dose (TCID_50_), reported as TCID_50_/mL for blood and swabs and TCID_50_/g for tissues, was calculated using the Reed–Muench method.

### Next-Generation Sequencing

Total RNA was extracted from whole blood or homogenized tissues, as described above and subjected to NGS. Briefly, RNA was converted to cDNA using the SuperScript™ IV VILO™ Master Mix (Invitrogen, USA) with the following modifications to the manufacturer’s protocol: Samples with CT values > 22 were diluted 1:10 in nuclease-free water; otherwise, samples were used neat. Following DNase treatment, reactions were immediately transferred on ice and not allowed to cool in the thermocycler. Reverse transcription reactions were completed using either a Proflex PCR system (Applied Biosystems, USA) or SimpliAmp Thermal Cycler (Applied Biosystems, USA) with the following modified thermocycling conditions: 25 °C for 10 min, 37 °C for 10 min, 50 °C for 10 min, 55 °C for 10 min, 65 °C for 10 min and 85 °C for 5 min. Next, cDNA was subjected to PCR amplification using the Phusion U Multiplex PCR Master Mix (Thermofisher Scientific, USA) with the following thermocycling conditions: 98 °C for 30 s followed by 30 cycles of 98 °C for 10 s, 65 °C for 30 s and 72 °C for 5 min. Amplicon sequencing of GPA-MARV was done using primers designed with primalscheme^34^ using Lake Victoria marburgvirus - Angola2005 strain Ang0754 (GenBank Accession No. DQ447659.1) as the reference sequence. Primers were designed according to the default parameters defined by primalscheme with amplicon sizes of 800 bp and overlap of 200 bp. PCR amplicons from each pool were purified using RNAClean XP (Beckman Coulter, USA) according to the manufacturer’s instructions and subsequently quantified on a Qubit Flex Fluorometer (Invitrogen, USA) using the 1X dsDNA BR Assay Kit (Invitrogen, USA). PCR amplicons from both pools were combined at equimolar concentrations, and library construction was performed using the Nextera XT DNA Library Preparation Kit (Illumina, USA), per the manufacturer’s protocol. Sequencing was done on a NextSeq2000 sequencer using a NextSeq 1000/2000 P1 Reagent Kit v3 (300 cycles) (Illumina, USA).

### Ferret Cytokine Luminex

Gamma irradiated serum samples from animals challenged with GPA-MARV, MA-MARV or GPA-EBOV were subjected to a Luminex Assay using the 12-plex ferret cytokine panel (Ampersand bio, USA) testing the following analytes IFNα, IL-2, IL-4, IL-6, IL-8, IL-12p40, IL-12p70, IL-17, MIP-1β, MCP-1, TNFα and IP-10. The Luminex assay was performed according to the manufacturer’s instructions, with the sole modification being that serum was clarified by centrifugation before the assay. Luminex plates were read using the MAGPIX Luminex system (Invitrogen, USA), and data collection, interpretation, and analysis were performed using the xPONENT software (Invitrogen, USA). Historical control sera from ferrets challenged with wild type EBOV (*n* = 2) and MARV (*n* = 2) from a previously published study^7^ were included to establish a baseline cytokine profile of ferrets challenged with wild type filoviruses.

### Data analysis

All RT-qPCR data was gathered and analyzed using the Quantstudio Design and Analysis Software (Applied Biosystems, USA). All graphs were generated using GraphPad Prism 9.5.1.

### Ethics statement

All animal work involving infectious filoviruses was conducted in the containment level-4 (CL-4) facility at the Canadian Science Center for Human and Animal Health of the Public Health Agency of Canada in Winnipeg, Canada. All experiments were reviewed and approved by the Animal Care Committee of the CSCHAH in accordance with guidelines from the Canadian Council on Animal Care. Animals were acclimatized for a minimum of 7 days prior to infection, given food and water ad libitum, and monitored daily. Environmental enrichment was provided throughout the study. All animals were anesthetized using isoflurane before conducting any procedures.

## Supplementary information

Supplementary Data1

Supplementary Data2

Supplementary Information

## Data Availability

All data are included within the published article and associated supplementary data files.

## Code availability

There is no custom code associated with submission.
