# Supplementary material for: Rodent adapted marburg viruses are lethal in ferrets
Source: Npj Viruses. 2025 Sep 8;3:67. doi: 10.1038/s44298-025-00147-4 (PMC12417545; doi:10.1038/s44298-025-00147-4)
Supplement: Supplementary file 1 — Supplementary Data1 [file 44298_2025_147_MOESM1_ESM.docx]

| **ANIMAL RECORD SHEET – FERRETS**  *Supplementary Information – Animal Scoring - Ferrets* | | | | | | | | | | | | | | |
| --- | --- | --- | --- | --- | --- | --- | --- | --- | --- | --- | --- | --- | --- | --- |
| **Investigator:** |  | | **AUD#:** |  | **Date:** |  | | **Animal ID#:** | | |  | | | |
| **Parameter** | **Degree** | | | | **DPI** | **28** |  | |  |  | |  |  |  |
|  |  |  |  |  | *Score* | *Score* | *Score* | | *Score* | *Score* | | *Score* | *Score* | *Score* |
| **Posture** | Normal | | | | 0 |  |  | |  |  | |  |  |  |
|  | Decreasing activity; decreasing normal behaviour; pilo-erection | | | | 3 |  |  |  |  |  |  |  |  |  |
|  | Huddled; not moving in cage | | | | 5 |  |  |  |  |  |  |  |  |  |
| **Temp.** | Hypothermic | ≤ 37.1^o^C ****euthanize**** | | | 25 |  |  | |  |  | |  |  |  |
|  | Decreased | 37.2 – 37.9^o^C ****consider euth.**** | | | 10 |  |  |  |  |  |  |  |  |  |
|  | Normal | 38.0 – 40.0^o^C | | | 0 |  |  |  |  |  |  |  |  |  |
|  | Elevated | 40.1 – 41.0^o^C | | | 2 |  |  |  |  |  |  |  |  |  |
|  | High | ≥ 41.1^o^C | | | 5 |  |  |  |  |  |  |  |  |  |
| **Weight Change** | Decrease ≥ 10% | | | | 10 |  |  | |  |  | |  |  |  |
| **Respiration** | Normal | | | | 0 |  |  | |  |  | |  |  |  |
|  | Increased or decreased | | | | 2 |  |  |  |  |  |  |  |  |  |
|  | Laboured; breathing through mouth | | | | 10 |  |  |  |  |  |  |  |  |  |
|  | Coughing or sneezing | | | | 2 |  |  |  |  |  |  |  |  |  |
| **Feces & Urine** | Normal consistency/volume; soft normal stool | | | | 0 |  |  | |  |  | |  |  |  |
|  | Feces absent or dry; decreased urine output; cloudy urine | | | | 2 |  |  |  |  |  |  |  |  |  |
|  | Wet/pasty; small, very dry stool; dark stool | | | | 2 |  |  |  |  |  |  |  |  |  |
|  | Liquid stool; blood in stool or urine; no urine >2x | | | | 10 |  |  |  |  |  |  |  |  |  |
| **Food & Water** | Normal eating/drinking | | | | 0 |  |  | |  |  | |  |  |  |
|  | Mildly decreased eating/drinking | | | | 1 |  |  |  |  |  |  |  |  |  |
|  | Moderately decreased eating/drinking | | | | 3 |  |  |  |  |  |  |  |  |  |
|  | Severely decreased eating/drinking | | | | 4 |  |  |  |  |  |  |  |  |  |
|  | Seriously decreased eating/drinking; refusing all food; dehydration apparent >2 days | | | | 10 |  |  |  |  |  |  |  |  |  |
| **Recumbent** | No symptoms | | | | 0 |  |  | |  |  | |  |  |  |
|  | Huddled on camera; active when cage opened | | | | 3 |  |  |  |  |  |  |  |  |  |
|  | Lies down but moves around | | | | 15 |  |  |  |  |  |  |  |  |  |
|  | Lies down and will not move | | | | 25 |  |  |  |  |  |  |  |  |  |
| **Attitude** | Normal | | | | 0 |  |  | |  |  | |  |  |  |
|  | Mildly depressed; responds to treats and toys | | | | 1 |  |  |  |  |  |  |  |  |  |
|  | Moderately depressed; response requires prodding; loses interest in treats and toys | | | | 3 |  |  |  |  |  |  |  |  |  |
|  | Severely depressed; no interest in treats; does not respond to human presence | | | | 10 |  |  |  |  |  |  |  |  |  |
| **Other** | Flushed appearance to skin | | | | 2 |  |  | |  |  | |  |  |  |
|  | Nasal discharge | | | | 2 |  |  | |  |  | |  |  |  |
|  | Visible rash | | | | 5 |  |  | |  |  | |  |  |  |
|  | Cyanosis | | | | 5 |  |  | |  |  | |  |  |  |
|  | Hemorrhage | | | Subcutaneous | 10 |  |  | |  |  | |  |  |  |
|  |  |  |  | Orifices | 15 |  |  |  |  |  |  |  |  |  |
| **TOTAL SCORE** | | | | | |  |  | |  |  | |  |  |  |
| *****Animal will be euthanized when a total score of 25 is reached*** | | | | | | | | | | | | | | |
| **BODY WEIGHT** | | | | | |  |  | |  |  | |  |  |  |
| **SCAN TEMP** | | | | | |  |  | |  |  | |  |  |  |
| **RECTAL TEMP** | | | | | |  |  | |  |  | |  |  |  |
