# Supplementary material for: Rodent adapted marburg viruses are lethal in ferrets
Source: Npj Viruses. 2025 Sep 8;3:67. doi: 10.1038/s44298-025-00147-4 (PMC12417545; doi:10.1038/s44298-025-00147-4)
Supplement: Supplementary file 2 — Supplementary Data2 [file 44298_2025_147_MOESM2_ESM.pdf]

Title: Rodent Adapted Orthomarburgviruses are Lethal in Ferrets

Authors: Zachary Schiffman[1,2], Lauren Garnett[1], Kaylie N Tran[1], Jonathan Audet[1], Kevin Tierney[1], Kim Azaransky[1], Shihua He[1], Logan Banadyga[1,2] and James E Strong[1,2, 3*]

Affiliations:

1) Special Pathogens Program, National Microbiology Laboratory Branch, Public Health Agency of Canada, Winnipeg, MB, Canada
2) Department of Medical Microbiology and Infectious Diseases, University of Manitoba, Winnipeg, MB, Canada
3) Department of Pediatrics and Child Health, University of Manitoba, Winnipeg, MB, Canada

Supplemental figures

Supplemental Table 1. RT-qPCR primer/probe sequences

Target	Forward Primer (5’-3’)	Reverse Primer (5’-3’)	Probe
MARV –L	GCAAAAGCATTCCCTAGTAACATGA<br>GCGAAGGCATTCCCTAGTAATATGA	CACCCCTCACTATRGCGTTYTC<br>CACCTCTTACTATGGCATTCTC	FAM-<br>TGGCACCAYAATTCAGCAAGCATAGG
EBOV - L	CAGCCAGCAATTTCTTCCAT	TTTCGGTTGCTGTTTCTGTG	FAM-<br>ATCATTGGCGTACTGGAGGAGCAG<br>FAM-<br>TCATTGGCGTACTGGAGGAGCAGG-

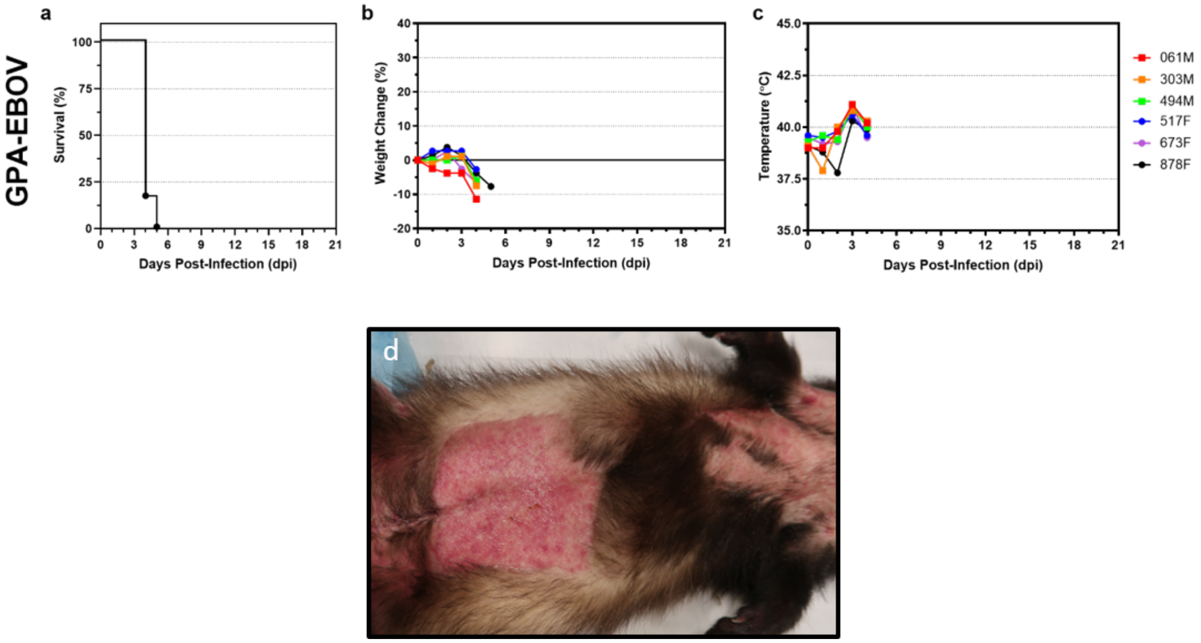

Supplemental Figure 1. Clinical parameters of ferrets inoculated with GPA-EBOV. Clinical parameters of ferrets (n=6 per group) inoculated with GPA-EBOV. Kaplan-Meier survival curves (**a**), percent weight change (**b**), microchip scan temperature (**c**). Representative image of a maculopapular rash observed at the terminal timepoint among GPA-EBOV challenged ferrets (**d**). Data from each animal are depicted as dots with individual animal IDs and sex (F/M) are indicated in the key.

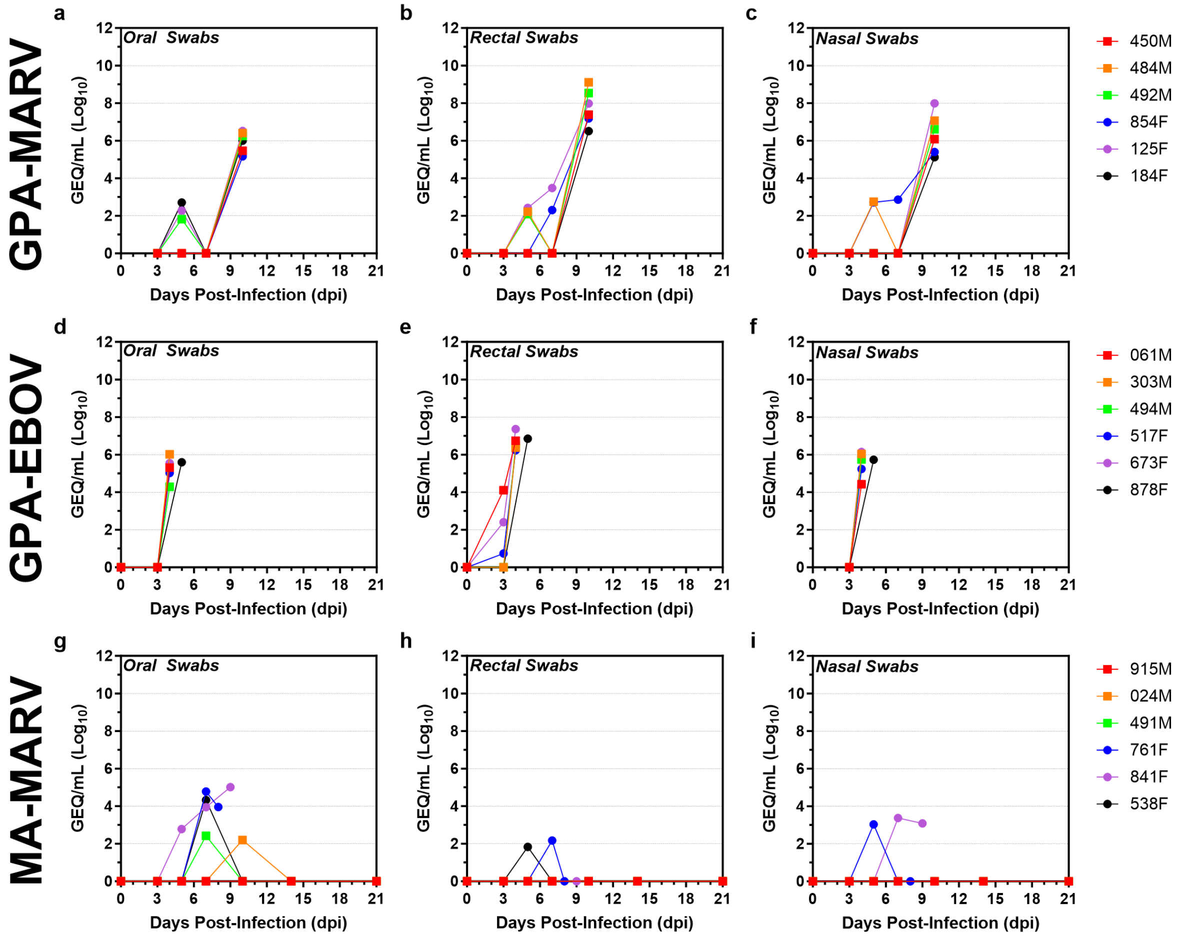

Supplemental Figure 2. Levels of viral RNA in oral, rectal and nasal swabs of ferrets infected with rodent adapted filoviruses. Oral (**a, d, g**), rectal (**b, e, h**) and nasal (**c, f, i**) swabs were collected from each animal challenged with GPA-MARV (**a-c**), GPA-EBOV (**d-e**) or MA-MARV (**g-i**) at 3, 5, 7, 10, 14 and 21 days post-infection (dpi) as well as at time of euthanasia to evaluate viral shedding by RT-qPCR. Results are depicted in genome equivalents per mL (GEQ/mL) for each animal and are depicted as dots with individual animal IDs, and sex (F/M) indicated in the key.

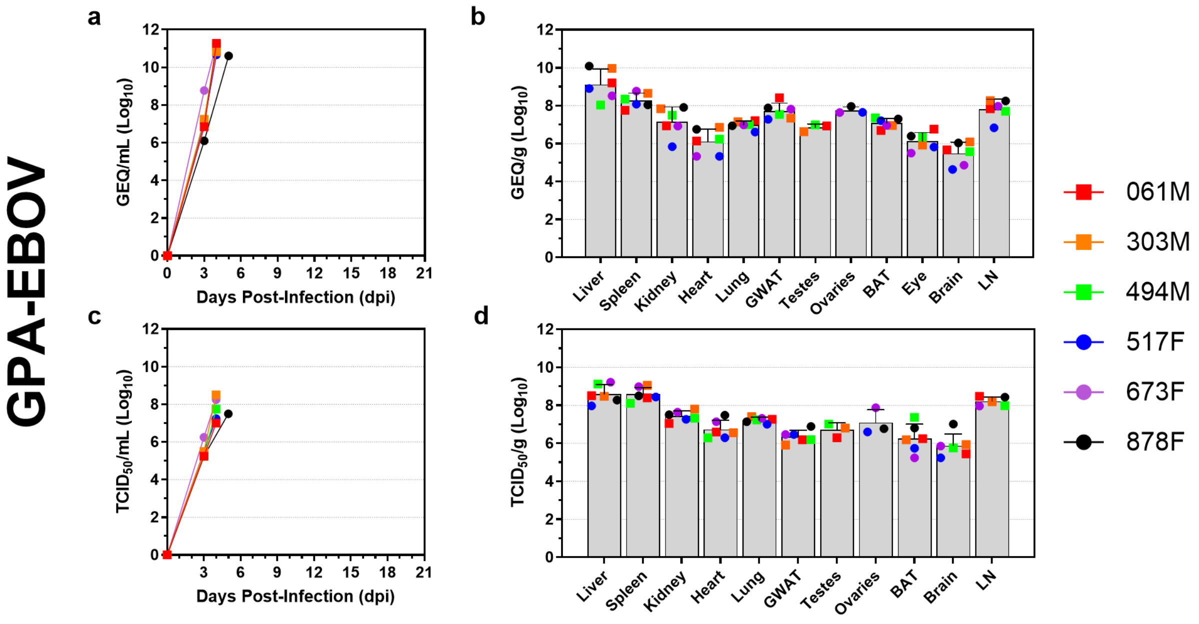

Supplemental Figure 3. Viral load in blood and tissues of ferrets infected with GPA-EBOV. Whole blood (**a, c**) was collected from each animal at 3, 5, 7, 10, 14 and 21 days post-infection (dpi) and at the time of euthanasia, while tissues (liver, spleen, kidney, heart, lung, gonadal white adipose tissue (GWAT), testes, ovaries, brown adipose tissue (BAT), eye, brain, and lymph node (LN)) were collected upon necropsy to evaluate viral loads among animals challenged with GPA-EBOV (**a-d**). Virus RNA was quantified by RT-qPCR (**a, b**) and infectious virus was quantified by $TCID_{50}$ (**c, d**). Results are depicted in genome equivalents per mL (GEQ/mL) for each animal (indicated by a square for males and circle for females) or mean tissue culture infectious dose per mL ($TCID_{50}$/mL) or per g ($TCID_{50}$/g) depicted as histograms, with data from each animal depicted as dots. Sex (F/M) are indicated the key.

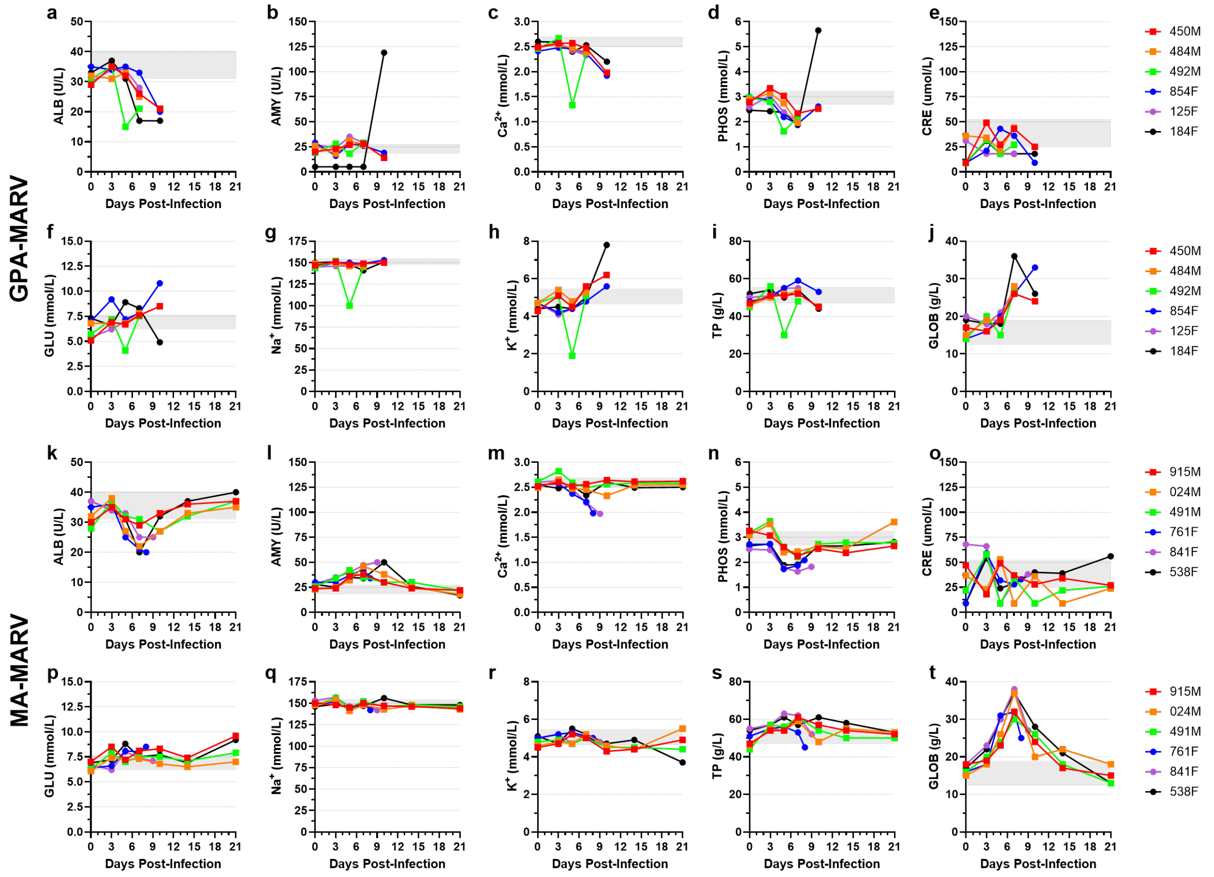

Supplemental Figure 4. Extended serum biochemistry of ferrets challenged with rodent-adapted orthomarburgviruses. Whole blood was collected from each animal on days 0, 3, 5, 7, 10, 14 and 21 days post-infection (dpi) as well as at time of euthanasia to evaluate serum biochemistry (**a-t**). Biochemistry analytes measured are as follows albumin [ALB]; amylase [AMY]; calcium [Ca^{2+}]; phosphorous [PHOS]; creatinine [CRE]; blood glucose [GLU]; sodium [Na^{+}]; potassium [K^{+}]; total protein [TP]; globulin [GLOB]. Individual animal IDs and sex (F/M) are indicated in the key.

GPA-EBOV Biochemistry

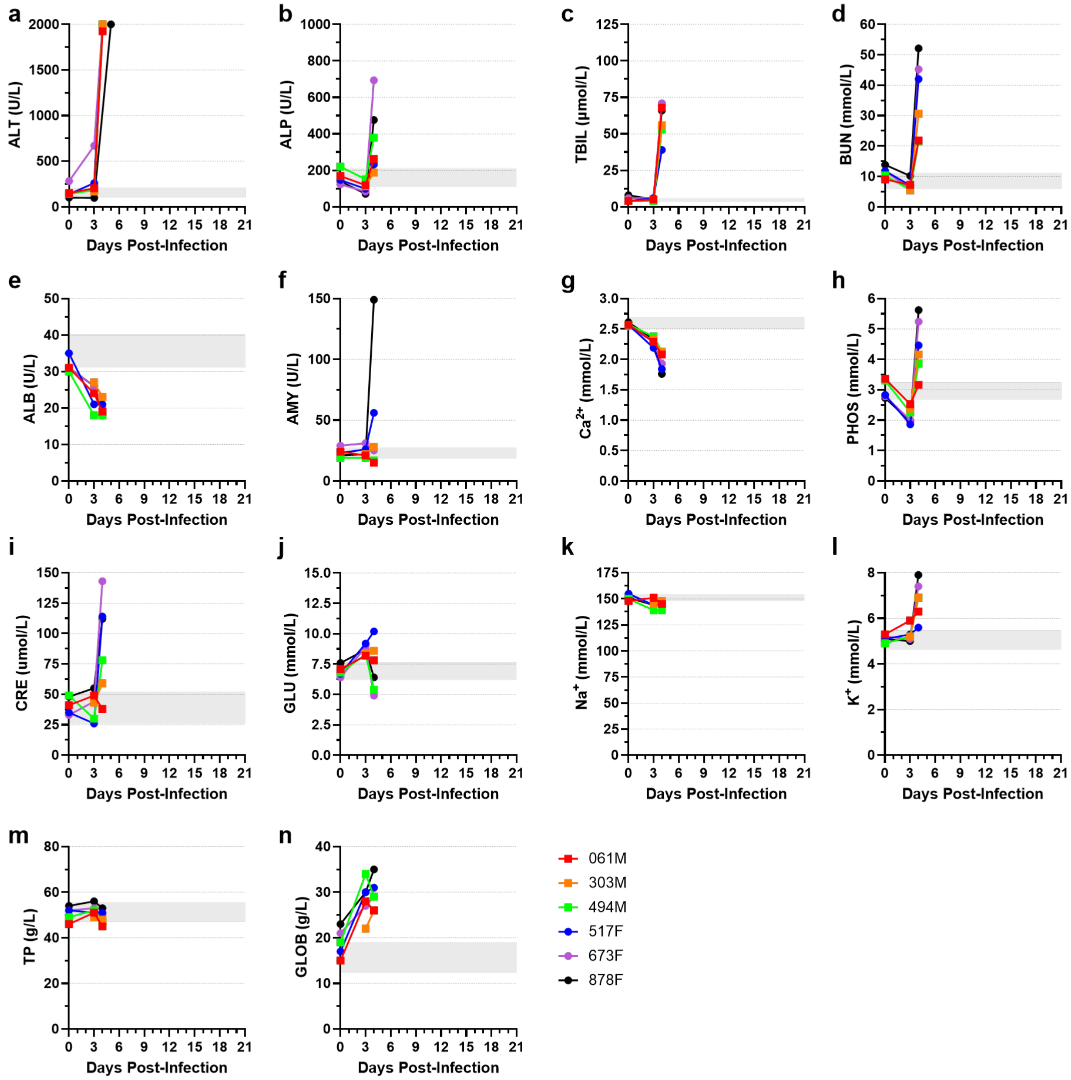

GPA-EBOV Blood counts

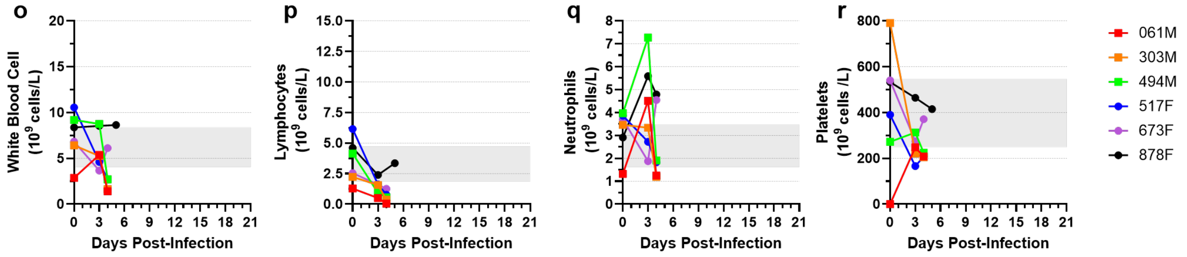

Supplemental Figure 5. Serum biochemistry and blood counts of ferrets challenged with GPA-EBOV. Whole blood was collected from each animal on days 0, 3, 5, 7, 10, 14 and 21 days post-infection (dpi) as well as at time of euthanasia to evaluate serum biochemistry (**a-n**) and complete blood counts (**o-r**). Biochemistry analytes

measured are as follows Individual animal IDs and sex (F/M) are indicated in the key. alanine aminotransferase [ALT]; alkaline phosphatase [ALP]; total bilirubin [TBIL]; and blood urea nitrogen [BUN]; albumin [ALB]; amylase [AMY]; calcium [Ca^{2+}]; phosphorous [PHOS]; creatinine [CRE]; blood glucose [GLU]; sodium [Na^{+}]; potassium [K^{+}]; total protein [TP]; globulin [GLOB]. Complete blood count analytes measured are as follows: white blood cells [WBC]; lymphocytes [LYM]; neutrophils [NEU]; platelets [PLT]. Individual animal IDs and sex (F/M) are indicated in the key.

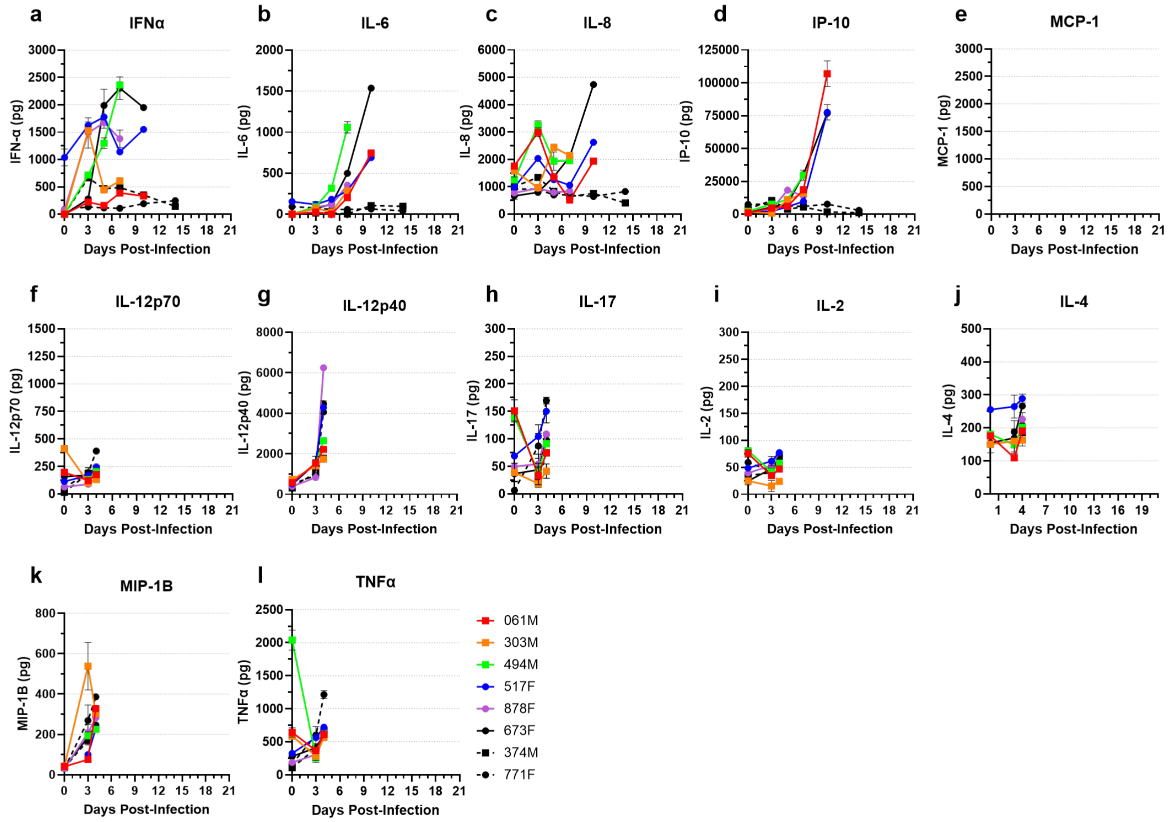

Supplemental Figure 6. The cytokine/chemokine response in ferrets challenged with GPA-EBOV. Serum was collected from each animal challenged with GPA-MARV (**a-e**), MA-MARV (**f-j**) at 0, 3, 5, 7, 10, 14 and 21 days post-infection (dpi) as well as at the time of euthanasia to evaluate cytokine/chemokine profiles using a 12-plex ferret Luminex assay. Analytes measured are as follows: Interferon-alpha [IFNα]; interleukin-6 [IL-6]; interleukin-8 [IL-8]; Interferon gamma-induced protein 10 [IP-10]; monocyte chemoattractant protein-1 [MCP-1]; Individual animal IDs and sex (F/M) are indicated in the key.

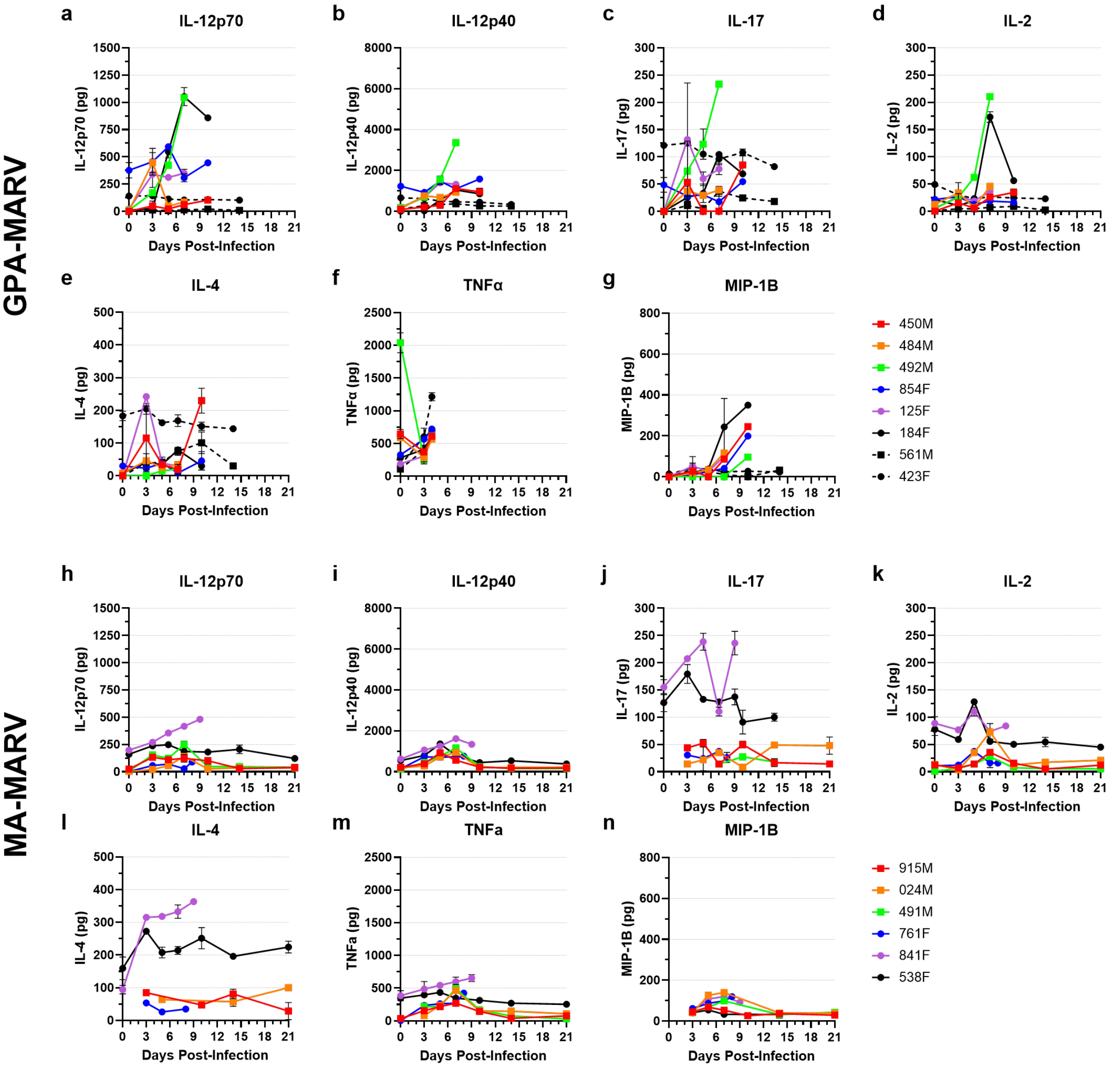

Supplemental Figure 7. Luminex assay on serum collected from ferrets challenged with rodent-adapted filoviruses (Extended)

Serum was collected from each animal challenged with (**A-G**) GPA-MARV, (**H-N**) GPA-EBOV or (**O-U**) MA-MARV at 0, 3, 5, 7, 10, 14 and 21 days post-infection (dpi) as well as at time of euthanasia to evaluate cytokine/chemokine profiles using a 12-plex ferret Luminex assay. Analytes measured are as follows: (**A, H, O**) interleukin-12 p70 [IL-12p70]; (**B, I, P**) interleukin-12 p40 [IL-12p40]; (**C, J, Q**) interleukin-17 [IL-17]; (**D, K, R**) interleukin-2 [IL-2]; (**E, I, S**) interleukin-4 [IL-4]; (**F, M, T**) monocyte chemoattractant protein-1 [MCP-1]; (**G, N, U**) macrophage inflammatory protein-1 beta [MIP-1B]. Individual animal IDs and sex (F/M) are indicated in the key.

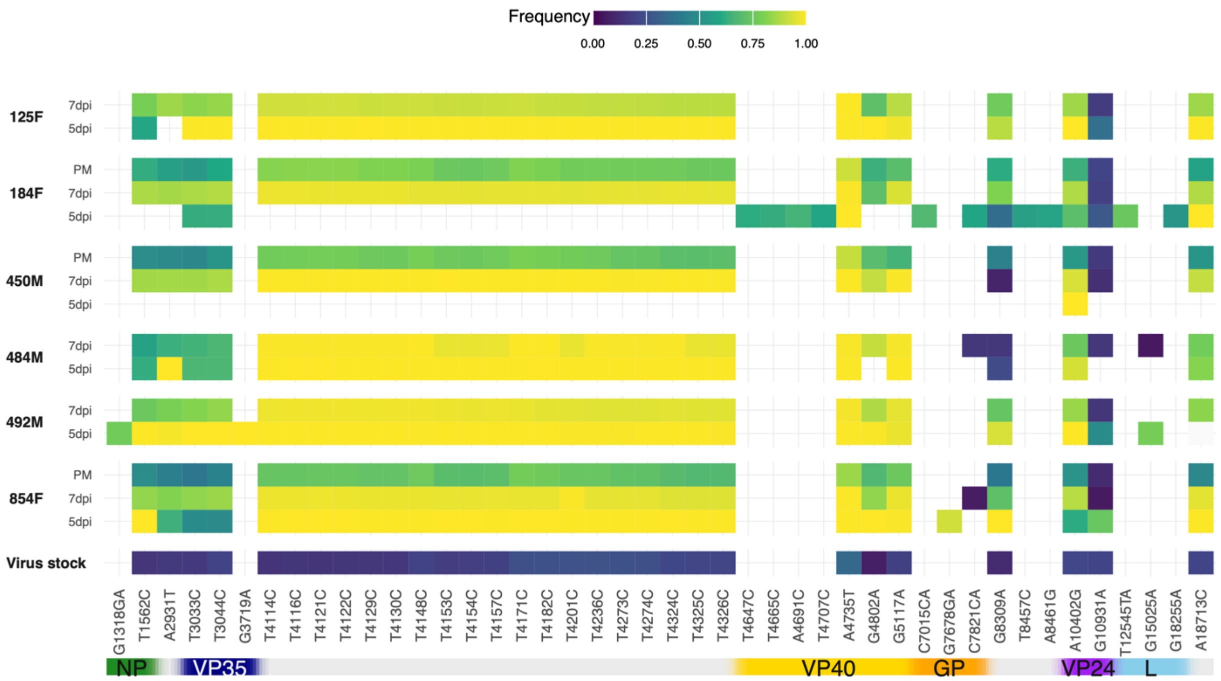

Supplemental Figure 8. Heatmap depicting mutations that meet consensus threshold in the blood of ferrets challenged with GPA-MARV

Whole blood was collected from ferrets challenged with GPA-MARV at 3, 5 and 7 days post-infection (dpi) as well as at time of euthanasia to track virus evolution overtime by Next-Generation Sequencing (NGS). Only mutations with a frequency of ≥ 50% that meet consensus are depicted within the heatmap. Virus stock corresponds to the GPA-MARV stock used for virus challenge. Mutations are denoted according to original base, position and mutant base. Note: no data available for blood at 3 dpi due to lack of viremia as well as PM for animals 492M, 125F and 484M due to lack of blood. Abbreviations: PM (postmortem), adenine (A), guanine (G), thymine (T), cytosine (C), nucleoprotein (NP), viral protein (VP), glycoprotein (GP), RNA-dependent RNA polymerase (L).

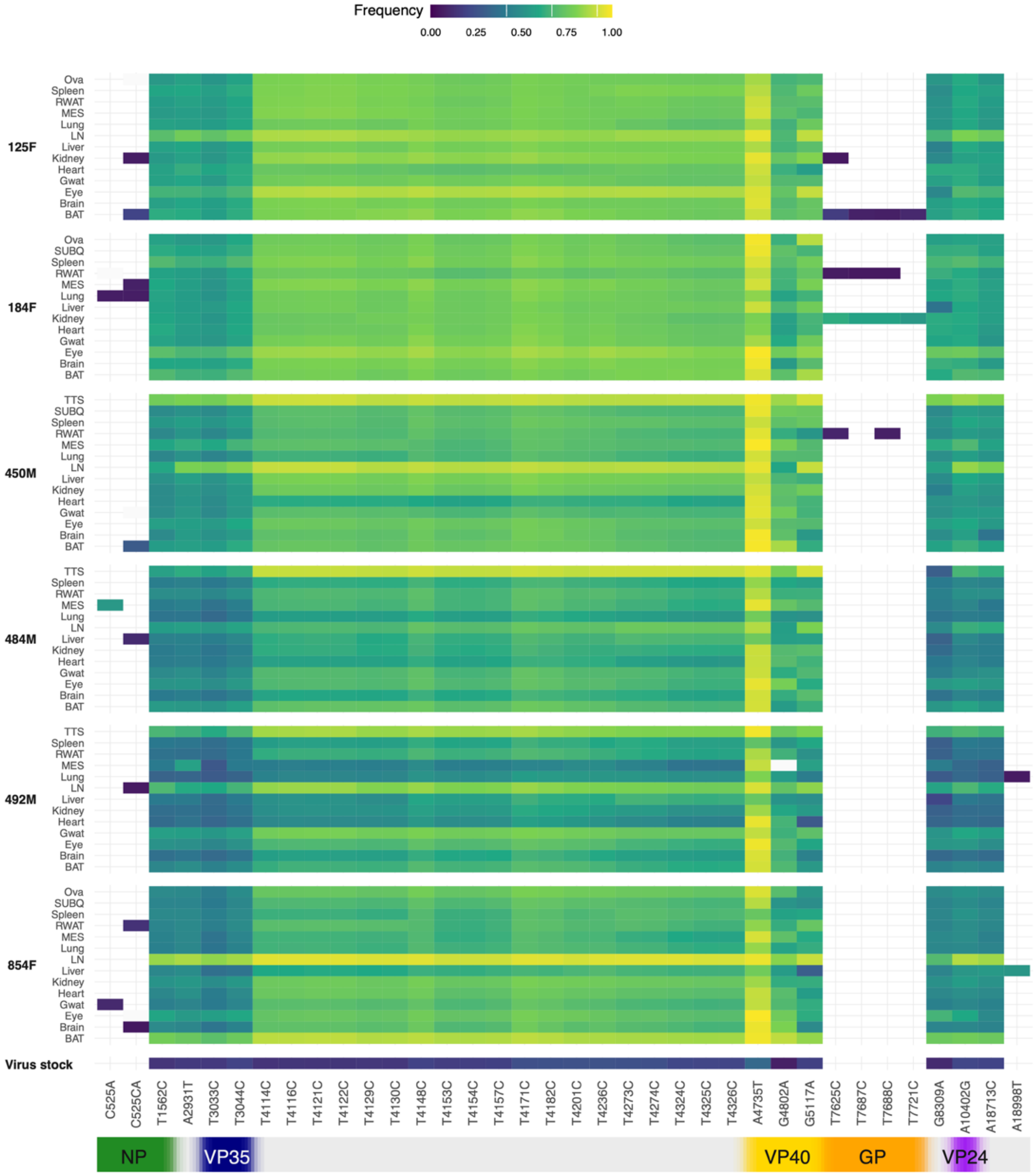

Supplemental Figure 9. Mutations that meet consensus threshold within tissues.

Tissues were collected from ferrets challenged with GPA-MARV at time of euthanasia to evaluate virus evolution across tissue by Next-Generation Sequencing (NGS). Only mutations with a frequency of ≥ 50% that meet consensus are depicted within the heatmap. Virus stock corresponds to the GPA-MARV stock used for virus challenge. Mutations are denoted according to original base, position and mutant base. Abbreviations: Ovaries (Ova), testes (TTS), subcutaneous white adipose (SUBQ), retroperitoneal white adipose (RWAT), mesenteric white adipose (MES), gonadal white adpipose (GWAT), lymphonode (LN), brown adipose tissue (BAT), adenine (A), guanine (G), thymine (T), cytosine (C), nucleoprotein (NP), viral protein (VP), glycoprotein (GP), RNA-dependent RNA polymerase (L).

Supplemental Figure 10. ANIMAL RECORD SHEET – FERRETS

**Investigator:**		**AUD#:**		**Date:**		**Animal ID#:**	

**Parameter**	**Degree**		**DPI**	**28**						
			Score	*Score*	*Score*	*Score*	*Score*	*Score*	*Score*	*Score*
**Posture**	Normal		0							
	Decreasing activity; decreasing normal behaviour; pilo-erection		3							
	Huddled; not moving in cage		5							
**Temp.**	Hypothermic	≤ 37.1ºC **euthanize**	25							
	Decreased	37.2 – 37.9ºC **consider euth.**	10							
	Normal	38.0 – 40.0ºC	0							
	Elevated	40.1 – 41.0ºC	2							
	High	≥ 41.1ºC	5							
**Weight Change**	Decrease ≥ 10%		10							
**Respiration**	Normal		0							
	Increased or decreased		2							
	Laboured; breathing through mouth		10							
	Coughing or sneezing		2							
**Feces & Urine**	Normal consistency/volume; soft normal stool		0							
	Feces absent or dry; decreased urine output; cloudy urine		2							
	Wet/pasty; small, very dry stool; dark stool		2							
	Liquid stool; blood in stool or urine; no urine >2x		10							
**Food & Water**	Normal eating/drinking		0							
	Mildly decreased eating/drinking		1							
	Moderately decreased eating/drinking		3							
	Severely decreased eating/drinking		4							
	Seriously decreased eating/drinking; refusing all food; dehydration apparent >2 days		10							
**Recumbent**	No symptoms		0							
	Huddled on camera; active when cage opened		3							
	Lies down but moves around		15							
	Lies down and will not move		25							
**Attitude**	Normal		0							
	Mildly depressed; responds to treats and toys		1							
	Moderately depressed; response requires prodding; loses interest in treats and toys		3							
	Severely depressed; no interest in treats; does not respond to human presence		10							
**Other**	Flushed appearance to skin		2							
	Nasal discharge		2							
	Visible rash		5							
	Cyanosis		5							
	Hemorrhage	Subcutaneous	10							
		Orifices	15							
**TOTAL SCORE**										

Animal will be euthanized when a total score of 25 is reached

**BODY WEIGHT**							
**SCAN TEMP**							
**RECTAL TEMP**							
